# Introducing leprosy post-exposure prophylaxis into the health systems of India, Nepal and Indonesia: a case study

**DOI:** 10.1186/s12913-017-2611-7

**Published:** 2017-09-29

**Authors:** A. Tiwari, L. Mieras, K. Dhakal, M. Arif, S. Dandel, J. H. Richardus, LPEP Study Group

**Affiliations:** 1 Department of Public Health, Erasmus MC, University Medical Center Rotterdam, Office Na 2219, Wytemaweg 80, 3015 CN Rotterdam, The Netherlands; 2 Netherlands Leprosy Relief, Amsterdam, The Netherlands; 3 Netherlands Leprosy Relief, Kathmandu, Nepal; 4 Netherlands Leprosy Relief, New Delhi, India; 5 Netherlands Leprosy Relief, Jakarta, Indonesia

**Keywords:** Leprosy, Health systems, Chemoprophylaxis, National Leprosy Control Programs

## Abstract

**Background:**

Leprosy has a wide range of clinical and socio-economic consequences. India, Indonesia and Nepal contribute significantly to the global leprosy burden. After integration, the health systems are pivotal in leprosy service delivery. The Leprosy Post Exposure Prophylaxis (LPEP) program is ongoing to investigate the feasibility of providing single dose rifampicin (SDR) as post-exposure prophylaxis (PEP) to the contacts of leprosy cases in various health systems. We aim to compare national leprosy control programs, and adapted LPEP strategies in India, Nepal and Indonesia. The purpose is to establish a baseline of the health system’s situation and document the subsequent adjustment of LPEP, which will provide the context for interpreting the LPEP results in future.

**Methods:**

The study followed the multiple-case study design with single units of analysis. The data collection methods were direct observation, in-depth interviews and desk review. The study was divided into two phases, i.e. review of national leprosy programs and description of the LPEP program. The comparative analysis was performed using the WHO health system frameworks (2007).

**Results:**

In all countries leprosy services including contact tracing is integrated into the health systems. The LPEP program is fully integrated into the established national leprosy programs, with SDR and increased documentation, which need major additions to standard procedures. PEP administration was widely perceived as well manageable, but the additional LPEP data collection was reported to increase workload in the first year.

**Conclusions:**

The findings of our study led to the recommendation that field-based leprosy research programs should keep health systems in focus. The national leprosy programs are diverse in terms of organizational hierarchy, human resource quantity and capacity. We conclude that PEP can be integrated into different health systems without major structural and personal changes, but provisions are necessary for the additional monitoring requirements.

**Electronic supplementary material:**

The online version of this article (10.1186/s12913-017-2611-7) contains supplementary material, which is available to authorized users.

## Background

Leprosy is an infectious disease, predominantly affecting peripheral nerves and the skin. It leads to a wide range of clinical symptoms, eventually resulting in disfigurement and disability if left untreated [1]. Additionally, the disease has severe socioeconomic consequences such as stigma and poverty, which may impact the patients and their families lifelong [2, 3]. The WHO calls to globally interrupt leprosy transmission and reduce grade-2 disabilities in newly detected cases to below 1 per million population by 2020 [4]. However, current progress indicates that these targets are difficult to achieve [5, 6]. In the year 2014, a total of 213,899 new cases were detected with a rate of 3.78 cases per 100,000 population. Southeast Asia accounted for 72% of the global new case load. India was the largest contributor (58.8%), followed by Brazil (14.5%) and Indonesia (8%). Nepal identified 3046 new cases in 2014, which is around 2% of the total Southeast Asia burden [5]. Hence, India, Indonesia and Nepal are important contributors to the global burden of leprosy despite established and relatively well-resourced control programs, and elimination of leprosy (zero incidence) needs alternative control strategies.

After integration, the general health systems are pivotal for leprosy service delivery. A health system is defined as *“the combination of resources, organization, financing and management that culminate in the delivery of health services to the population”* [7]. Early case detection and subsequent treatment with multi-drug therapy (MDT) are the key strategies to reduce the disease burden [8, 9]. Health systems however, do not appear to be efficient in detecting cases early, as the grade 2 disability rate remained stable (between 0.23 to 0.25 per 100,000 population) over the last 10 years [5]. Furthermore, the stagnation in the new case detection rate (NCDR) and relatively high child case rates in many countries indicate that transmission of *Mycobacterium leprae*, the causative agent of leprosy, is ongoing and that current methods, including MDT, are insufficient to break transmission [10, 11]. The transmission of the *M. leprae* bacteria is complex poorly understood [12, 13]. Also, it has been argued that leprosy programs are not implemented properly [6, 14], and needs to be improved [15, 16].

There is sufficient evidence that chemoprophylaxis with Single Dose Rifampicin (SDR) is efficacious in reducing the risk of developing leprosy among contacts of leprosy patients [17, 18]. It has thus been recommended to assess the effectiveness of SDR in different field settings [19]. Therefore, the Leprosy Post-Exposure Prophylaxis (LPEP) program was initiated by different stakeholders in close collaboration with the ministries of health of eight countries - India, Nepal, Indonesia, Myanmar, Sri Lanka, Tanzania, Brazil and Cambodia. LPEP activities started in 2014 for a duration of three years. The objective of LPEP is to assess the impact on the new case detection rate, measured through strengthened surveillance and reporting systems and its feasibility in diverse routine programme settings. The program has three prime components: Contact tracing; screening; and SDR administration. It is designed to complement and be integrated into the national leprosy control programs, rather than operating vertically. Moreover, it aims to contribute to the strengthening of the general health care systems by providing support in human resources, training and program monitoring.

The primary objective of this work is to compare national leprosy control programs and adapted LPEP strategies in India, Nepal and Indonesia. The secondary objective is to summarize the lessons learned during the first year of implementation.

## Methods

### LPEP program sites

In India, the program is operating in the union territory (UT) of Dadra and Nagar Haveli (DNH), situated in the west of India between the state of Gujarat and Maharashtra. Nepal is implementing the program in the *tarai* (plains) districts of Jhapa, Morang and Parsa. All three districts share boundaries with India. In Indonesia LPEP is implemented in Sumenep district, which is a regency of East Java province, situated on the eastern end of Madura Island. All intervention areas are high leprosy endemic and have been selected based on the recommendations of the respective ministry of health (Table 1).

**Table 1:** Demographic, geographical and epidemiological profile (2015–16) of the LPEP program sites

Country (2015–16)	India	Nepal			Indonesia
Sub-national area	Dadra & Nagar Haveli, UT	Jhapa District	Morang District	Parsa District	Sumenep District
Population	427,462	887,023	1,044,071	660,249	1,059,000
Area (km^2^)	491	1606	1855	1353	1998
New cases detection rate (NCDR/100,000)	99.4	20.97	19.3	16.56	43.3
Percent new cases of MB leprosy	26.5	60.75	49.0	41.44	76.3
Percent new cases with DGII	1.8	2.69	1	NA	5.5
Percent new cases:					
- Females	57.8	46.24	44	25.22	46.2
- Children	23.2	3.76	8.9	5.40	6.5

*UT* Union Territory, *NA* Information not available, *NCDR* New Case Detection Rate, *MB* Multi Bacillary, *DGII* Disability Grade II

### Study design

The study followed the multiple-case study design with single units of analysis [20]. The case study methodology was selected because it was suitable for the objective of the research, i.e. comparing LPEP (case) in the context of the national leprosy control programs. Furthermore, the selected methodology enables exploratory analysis by using data from multiple sources. The study aims to cover a broader range of complex field conditions that have a role in developing LPEP strategies in each country.

### Data collection

We collected quantitative and qualitative data. The data collection methods were direct observations (facility and service delivery), interviews (open-ended and semi-structured conversations) with the staff at various levels, and desk review. The type of information (online and printed) reviewed were peer reviewed publication, department reports, and other program documents such as guidelines, training manuals and annual report.

The study was divided into two phases (Table 2). In the first phase, we reviewed the national leprosy control programs in the three countries. The first set of data was collected through desk review, followed by a field visit in each country between April 2015 and January 2016. The desk review aimed to identify documents describing the standard operating procedures and policies of the national leprosy programs, whereas the objectives of the field visits were to interview staff and observe on-site activities. During field visits, we collected relevant documents that were not available online. The staff at the national, provincial and field level were interviewed to assess perceived reasons behind current epidemiological trends, and to describe their routine practices and associated challenges. An additional file shows this in more detail [see Additional file 1]. Furthermore, we verified the standard operating procedures and data trends published by the national programs during interviews. The first phase data were then used to assess and compare the different national programs and describe a baseline for LPEP.

**Table 2:** Details of the data collection methods, data type and sources

Data Collection Method	Type of data and sources	Nature
Phase I: National Leprosy Programs		
Desk review	Secondary data from scientific papers, archival records and document on national leprosy control programs	Quantitative data on the epidemiology and performance of the programs. Qualitative data on the SOP and policies
Direct observation	Primary data	Qualitative observations of the activities such as contact tracing, treatment rehabilitation, etc.
Interviews	Primary data	Qualitative data on explanations of epidemiological trends, routine functioning, challenges and solutions
Phase II: LPEP Program		
Desk review	Secondary data on LPEP service delivery from MIS	Quantitative data on the coverage of services
Direct observation	Primary data	Qualitative observations of the LPEP activities such as screening, SDR distribution and recording & reporting
Interviews	Primary data	Qualitative data on LPEP routine functioning, challenges and solutions

*SOP* Standard Operating Procedures, *MIS* Monitoring Information System

In the second phase, we reviewed the LPEP activities at study site level, i.e. UT of Dadra and Nagar Haveli in India, Morang and Jhapa districts of Nepal, and Sumenep district of Indonesia. We visited each country twice between April and November 2015, after the inception of LPEP. The data collection methods were identical with the ones used in the first phase. Quantitative data were mainly related to the program coverage. The qualitative data were collected on the LPEP implementation practices. We focused on the difference between planned and actual implementation [21]. The health staff were interviewed to describe LPEP practices for various activities such as SDR distribution, contact tracing, screening, recording and reporting. The focus was on the coordination and integration of activities with the national leprosy programs. Finally, respondents were asked about the challenges faced during the pilot. A special focus was on the anticipated integration of PEP into the national programs.

### Data analyses

**Fig. 1:**
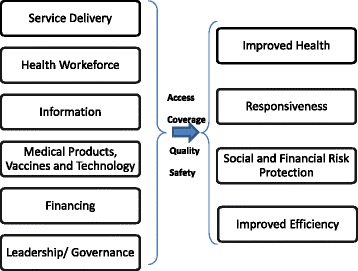
The WHO health system building blocks framework (2007)

The national leprosy control programs are part of the general health care system, and LPEP is integrated into it. Therefore, we adopted the WHO health system framework [22, 23] to outline the main components of the health system, as presented in Fig. 1.

These components were elaborated by the common emerging themes, identified from the primary and secondary data from the first phase and second phase.

We used the epidemiological (quantitative) data to assess the leprosy situation and the (qualitative) data on implementation to depict the program and LPEP project functioning respectively. The qualitative data on standard operating procedures and actual implementation were verified to minimize bias and assess similar patterns.

## Results

### National Leprosy Control Programs

**Fig. 2:**
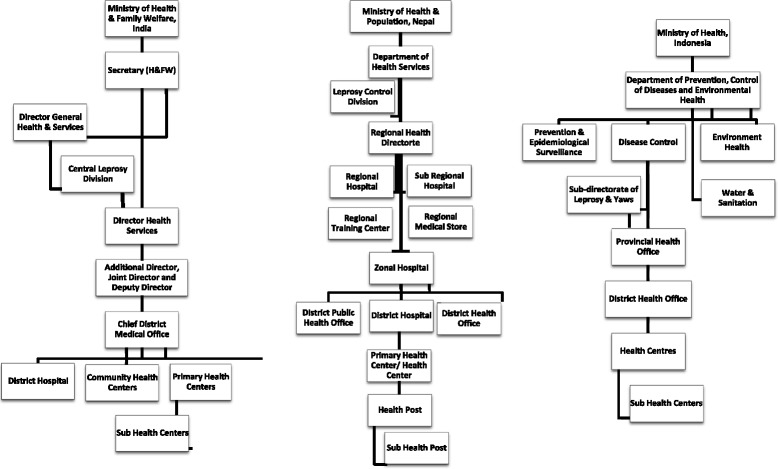
Organogram of the Health Services in India, Nepal and Indonesia

The general health care system is based on a three-tier structure in all reviewed LPEP countries, i.e. national, provincial and district level (Fig. 2).

The Indian leprosy program is called the National Leprosy Elimination Program (NLEP), whereas the Nepal and Indonesian programs are indicated as National Leprosy Control Program (NLCP). An additional file lists the official leprosy control/elimination strategies [see Additional file 2]. The leprosy control programs are operational throughout the countries, however, special attention is given to the high endemic areas. Case detection is mainly passive, although India and Indonesia reported instances of outreach leprosy activities, integrated or non-integrated with other diseases. The periodicity and focus of such activities (only in high endemic areas) is not fixed, and varies depending on the local situation and available means. Contact tracing was already a part of all reviewed leprosy programs before LPEP, but in practice only household contacts were covered in all three countries. The programs in Nepal and Indonesia depend largely on the paramedical staff located on the peripheries. The role of doctors is limited to the confirmation of unclear cases and management of complicated cases at higher levels. The presence and support of volunteers is strongest in India as compared to the other countries. Volunteers are actively engaged in information dissemination, suspect identification, and monitoring treatment adherence. The comparative details of national programs are listed in Table 3.

**Table 3:** Description of National Leprosy Control/Elimination Programs in India, Nepal and Indonesia based on WHO framework

WHO Framework	Themes	NLEP India	NLCP Nepal	NLCP Indonesia
Service Delivery	Coverage (Prevalence) 2014	88,833 cases registered and treated (Source: Global leprosy update 2014)	2382 cases registered and treated (Source: Global leprosy update 2014)	19,949 cases registered and treated (Source: Global leprosy update 2014)
	Infrastructure	153,655 Sub Center; 25,308 PHCs; 5396 CHCs (Source: Rural Health Statistics 2015, India)	208 PHCs; 1559 HPs; 2643 SHP (Source: Annual Report 2013–14, Dept. of Health, Nepal)	3395 HCs with IPD and 6345 HCs with only OPD (Source: Jumlah Puskesmas 2015, Indonesia)
	Activities	Case detection is mainly passive with few periodic active outreach	Case detection is mainly passive	Case detection is mainly passive with few periodic active outreach
		Routine household contact tracing	Routine household contact tracing	Routine household contact tracing; integrated SDR since 2012 in two districts
		Suspect identification & their adherence is checked by volunteers (ASHA) at field level	Suspect identification & their adherence is checked by volunteers (FCHV) at field level	Suspect identification & their adherence is checked by paramedical staff (village midwife)
		Contact screening by paramedical staff (PMW/ANM) at sub-center	Contact screening by paramedical staff (Leprosy Focal Person) at Health Post	Contact screening by paramedics staff (Leprosy officer) at HC
		Confirmation diagnosis by doctor at PHC and higher	Confirmation diagnosis by Leprosy focal person / doctor at Health Post and higher	Confirmation diagnosis by Leprosy officer at HC and doctor at higher level
	Process	Refer Fig. 3		
	MDT supply (Source: Interviews)	No stock out situation reported at peripheral level	Seldom stock out situation reported for a very short period at peripheral level	A major stock out situation reported in 2016 at peripheral level
Health Workforce	Staff	General health care staff. High epidemic PHCs have additional staff	General health care staff	General health care staff
	Leprosy Training	10,624 Doctors, 24,255 Paramedics and 104,011 volunteers trained on leprosy (Source: NLEP Progress Report 2014–15)	150 health worker trained on leprosy. (Source: Annual Report 2013–14, Dept. of Health, Nepal)	120 Doctors, 516 leprosy staff trained on leprosy in 2014 (Source: Subdit Kusta 2014, Indonesia)
Information	Indicators	Standard set of indicators as per WHO	Standard set of indicators as per WHO	Standard set of indicators as per WHO
	Data Management	Individual at sub-center level, then aggregated.	Individual at health-post level, then aggregated. General MIS electronic entry at district level but limited leprosy indicators.	Individual at sub-center level, then aggregated
	Supervision & Monitoring	CLD State Leprosy Office & District Leprosy Officer	CLD, Regional Health Directorate and District Health / Public Health officer	Department of Leprosy & Yaws (central), Provincial Leprosy Office and District Health Office
	Reporting	Monthly, quarterly and Annually. Bottom-up at all levels	Monthly, quarterly and Annually. Bottom-up at all levels	Monthly, quarterly and Annually. Bottom-up at all levels
Innovation	New initiatives	Developed *M.w* vaccine	NA	NA
Financing	Budget	NLEP total budget decreased by 9.8% from 2014 to 15 to 2015–16 (Source: MoHFW, Outcome Budget 2014–15 & 2015–16)	NLCP recurrent budget (released) was increased by 58% from 2012 to 13 to 2013–14 (Source: Annual Report Dept. of Health, 2012–13 & 2013–14)	NA
	Funding	CLD and State Leprosy Office	Ministry of Health and Population	Sub-directorate Leprosy & Yaws and District Health Office
	OOPs in leprosy	No evidence		
	Periodicity of funds (Source: Interviews)	Sometimes delay in salary disbursement at peripheral level or case reimbursements to ASHA	Sometimes delay in salary disbursement at peripheral level or case reimbursements to FCHV	Mostly on time
Governance	National Strategy	Strategy focus on decentralization of leprosy services. For more information, refer Additional file 1	Strategy focus on disability and rehabilitation. For more information, refer Additional file 1	Strategy focus on early detection. For more information, refer Additional file 1
	Organization structure	Fig. 2		
	Integration	Integrated into general health system	Integrated into general health system	Integrated into general health system

*ANM* Auxiliary Nurse Midwife, *ASHA* Accredited Social Health Activist, *CHC* Community Health Center, *CLD* Central Leprosy Division, *FCHV* Female Community Health Volunteer, *HC* Health Center, *HP* Health Post, *LFP* Leprosy Focal Person, *MPW* Multipurpose Worker, *NA* Not Available, *PHC* Primary Health Center, *PMW* Para Medical Worker, *SHP* Sub-Health Post

**Fig. 3:**
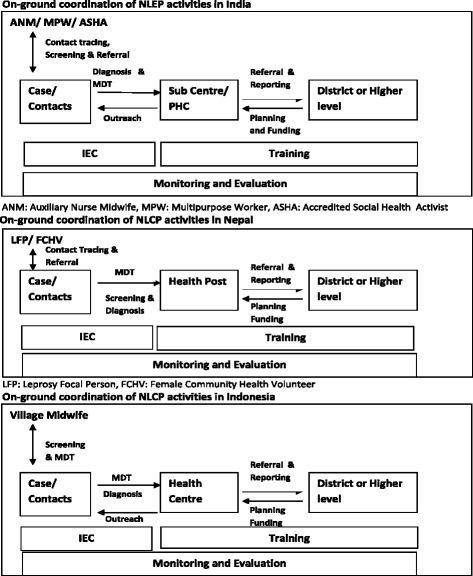
Diagram illustrating the implementation process under the National Leprosy Control / Elimination Programs in India, Nepal and Indonesia

The overall implementation process and the coordination between different staff levels is comparable in the three countries (Fig. 3).

**Fig. 4:**
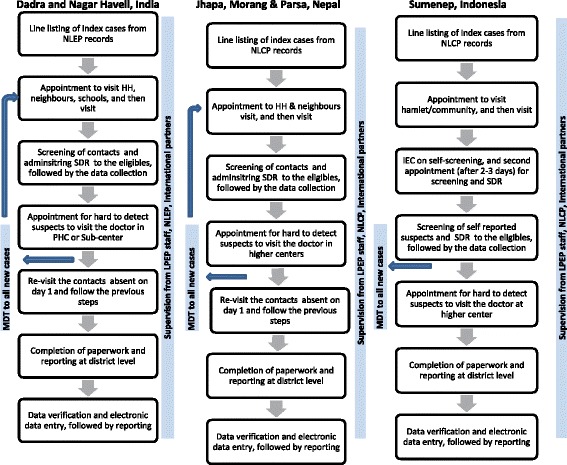
Flow chart of LPEP activities in India, Nepal and Indonesia

Service delivery is integrated into the general health care system in all three countries. However, central leprosy divisions have an extensive role in planning, funding and monitoring. The Indonesian health system is the most decentralized in terms of higher autonomy of districts in planning and allocating funds between diseases or activities. Next, MDT supply is based on the demand, i.e. case load of health facilities. Mostly the supply chain is smooth, but short periods of out-of-stock instances were reported from peripheral centres in Nepal. The general health care staff are involved in the implementation of the leprosy program, but in India high endemic districts occasionally receive top-up human resource budgets under the NLEP. In all countries training is the shared responsibility of provincial and district health departments. The recording and reporting includes all the indicators prescribed by WHO to estimate the burden [5, 8, 9]. Other reported indicators are on coverage of services, which varies between countries due to difference in activities. Nepal has developed an electronic database portal named WeBLeRS, capable of individual level data entry. Unfortunately, WeBLeRS is only used in a limited number of high endemic districts. Remaining countries are recording individual data on paper which remains at the field level. Subsequently, the aggregated data is reported to higher levels. Supervision and reporting follow the same structure and periodicity in all the three countries (Figs. 3 and 4).

### LPEP inception and target population

The LPEP field activities started in March 2015 in India, covering retrospective cases and contacts of the last two years. In Nepal, LPEP was slightly delayed due to the earthquake on 25 April 2015, therefore field implementation started in May 2015, covering retrospective cases and contact of the last one year. In Indonesia LPEP field implementation started in January 2015, with no target to cover retrospective cases. Instead, all leprosy cases diagnosed since January 1st 2015 are aimed to be covered, excluding the cases of 8 health centres, located on the remote islands of that regency in Indonesia. These islands are hard to reach and accessibility is limited.

### LPEP implementation comparison

The LPEP service delivery in all three countries is fully integrated into the general health care systems. Indonesia is practicing extended contact tracing using self-screening, whereas in India and Nepal the contacts are screened by paramedical staff. In the case of self-screening, the first field visit is dedicated to Information Education and Communication (IEC) on self-screening, followed by a second field visit (after 2–3 days) for investigation of self-reported suspects and SDR administration (Fig. 4).

In India, contacts are defined as Household, Neighbours and Social contacts (only school class fellows), whereas in Nepal and Indonesia only Household and Neighbours contacts are included. The minimum age to provide SDR is 2 years in all the three countries. Common activities include line listing, contact tracing, screening, SDR administration, recording, reporting and monitoring (Fig. 4). Rifampicin is procured by the local Department of Health in all the three countries. LPEP appointed staff in India are LPEP supervisor (*n* = 1) and research assistants (*n* = 4). The Nepal program is supported by a LPEP manager (n = 1) and district supervisors (*n* = 3), whereas Indonesia appointed only a LPEP manager (n = 1). The staff dedicate their full time to conduct training, supervision, assistance and reporting. All LPEP staff were trained in Training of Trainers (ToT) just before the program field implementation. Two types of trainings were imparted in all three countries, i.e. operational training and data management training. The LPEP data is collected on paper forms in the field, and reported to district level where electronic data entry takes place. A similar Microsoft Access database is used in all three countries, which collects demographic, epidemiological, clinical and coverage indicators. The supervision and monitoring structure is similar in all countries. Furthermore, Indonesia distributes IEC hand fans and packed drinking water during leprosy activities. The NGO funds are reported to be disbursed on time, however the government disbursements are aligned with the national program’s schedule. The comparative details of LPEP are listed in Table 4.

**Table 4:** Description of LPEP country programs in India, Nepal and Indonesia based on WHO framework

WHO Framework	Themes	LPEP Dadra and Nagar Haveli, India	LPEP Morang, Jhapa and Parsa, Nepal	LPEP Sumenep, Indonesia
Service Delivery	Average coverage (2015–16)	SDR coverage is average 22 contacts per index case	SDR coverage is average 23 contacts per index case	SDR coverage is average 33 contacts per index case
	Infrastructure	General health care system	General health care system	General health care system
	Activities	Line listing of HH, Neighbours and social contacts	Contact tracing of HH and Neighbours	Contact tracing of HH and Neighbours
		HH, neighbours and school visits by volunteers (ASHA) and paramedics (ANM/PMW)	HH and neighbours visits by volunteers (FCHV) and paramedics (LFP)	Community gathering by village midwife and paramedics (LO)
		Individual screening of contacts by paramedics	Individual screening of contacts by paramedics	Self-screening and then re-screening of the suspects by paramedics
		SDR distribution immediately after screening	SDR distribution immediately after screening	SDR distribution after 2–3 days of IEC on self-screening
		Onsite data collection (paper forms)	Onsite data collection (paper forms)	Onsite data collection (paper forms)
	Process	Refer Fig. 4	Refer Fig. 4	Refer Fig. 4
	SDR supply	Rifampicin is procured by Dept. of Health in al dosages. Syrups available	Rifampicin is procured by Dept. of Health in all dosage. Syrups not available	Rifampicin is procured by Dept. of Health in all dosage. Syrups not available
Health Workforce	Staff	General health care staff + LPEP Supervisor (1) and Research assistants (4)	General health care staff, + LPEP Manager (1) and District supervisors (3)	General health care staff + LPEP manager (1)
	Training	LPEP operations and data management training to the staff before inception	LPEP operations and data management training to the staff before inception	LPEP operations and data management training to the staff before inception
Information	Indicators	Demographic, Epidemiology, Clinical and coverage indicators	Demographic, Epidemiology, Clinical and coverage indicators	Demographic, Epidemiology, Clinical and coverage indicators
	Data Management	Electronic data entry at district level by RAs in standard database (similar in all countries)	Electronic data entry at district level by SAs in standard database (similar in all countries)	Electronic data entry at district level by DLO in standard database (similar in all countries)
	Supervision	Filed supervision by LPEP staff (daily bases), National program (periodic), International partners (twice a year)	Filed supervision by LPEP staff (daily bases), National program (periodic), International partners (twice a year)	Filed supervision by LPEP staff (daily bases), National program (periodic), International partners (twice a year)
	Reporting	Monthly, quarterly and Annually. Bottom-up at all levels	Monthly, quarterly and Annually. Bottom-up at all levels	Monthly, quarterly and Annually. Bottom-up at all levels
Innovation	Initiatives	Rifampicin available in syrup for pediatric cases	No initiatives identified	Hand fan with leprosy and self-screening information.
Financing	Funding	Majorly Govt. funds. NGO funding only for LPEP staff, monitoring and trainings	Majorly Govt. funds. NGO funding only for LPEP staff, monitoring and trainings	Majorly Govt. funds. NGO funding only for LPEP staff, monitoring and trainings
	Funds disbursement	On time disbursement of NGO funds. The government funds disbursement depends on national program’s status	On time disbursement of NGO funds. The government funds disbursement depends on national program’s status	On time disbursement of NGO funds. The government funds disbursement depends on national program’s status
Governance	Strategy	Extended contact tracing, including social contacts (school children)	Extended contact tracing	Extended contact tracing with self- screening
	Integration	Integrated into general health system	Integrated into general health system	Integrated into general health system

*ANM* Auxiliary Nurse Midwife, *ASHA* Accredited Social Health Activist, *DLO* District Leprosy Officer, *FCHV* Female Community Health Volunteer, *HH* Household, *IEC* Information Education Communication, *LFP* Leprosy Focal, *LO* Leprosy Officer, *NGO* Non-governmental Organization, *PMW* Multipurpose Worker, *RA* Research Assistant, *SA* Statistical Assistant, *SDR* Single Dose of Rifampicin

### Challenges in the first year of implementation

The initial months of the program field work were characterized by intense activities, due to the recruitment of retrospective leprosy cases. The country programs have been implemented by the general health care staff, after striking a balance between LPEP and other disease programs. The most common problem reported by the staff was the additional data collection work load (especially, filling of consent forms of cases and contacts) due to the research nature of the program. Next, not all contacts are present on the screening day, therefore health staff need to visit 2–3 times to achieve optimal coverage. Participation of male contacts is lower compared to females because they are more often out of the home to work. According to the field staff, refusals are more common in urban areas than rural areas, probably due to stigma, therefore more efforts reported to be deployed in urban areas to explain the program and the significance of SDR. A particular challenge is that houses are often dark while good light is required for screening, but females cannot be screened in the open.

## Discussion

The general health care system is the covering umbrella of leprosy services, thus we emphasize that all field-based leprosy research should be aligned with the local health system realities. The above statement is more relevant in a post global elimination scenario, when resources are reduced, but the pressure is high to deliver pragmatic results [24]. Correspondingly, feasibility also depends on the capacity of the health systems to accommodate new interventions. Systematic and sustained health system strengthening is important. Continuous and coordinated efforts are needed from various components (including disease specific programs) of a health system [25, 26]. For example, the coordination between leprosy and TB departments is desired to collectively deal with the risk of rifampicin resistance and to ensure proper follow-up of suspected TB cases identified in the frame of leprosy screening [27, 28]. Furthermore, leprosy service delivery also experiences common limitations of a weak health system such as poor accessibility, availability, affordability and quality [29–31]. Despite that the cross cutting evidence on leprosy and health systems is limited. As an exception, integration of vertical leprosy programs into the general health care system is a well-documented topic [32]. Most of the experiences however, are in the form of commentaries on individual cases. We recommend to synthesize the available literature on integration in a systemic way to highlight the differences and derive a framework, which can be further developed into a standardize tool to measure the level of integration. This is relevant because leprosy programs are partially integrated in many countries and such a tool can help in measuring performance over time. Further, the framework can be applied to other vertical programs.

The London Declaration recommends to increase funding for leprosy and other Neglected Tropical Diseases (NTDs) [4]. However, funding continues to decline, e.g. the total budget of NLEP India was decreased by 9.8% from 2014 to 2016 [33, 34]. Besides public funding, national leprosy programs should also promote inclusion of their services into other financial risk protection schemes [35]. In many high epidemic countries, state run insurance schemes are operational [36]. The leprosy programs should strive for a high coverage of their target population under such schemes, as leprosy poses a high financial risk [37].

Our study showed that the national leprosy programs as part of the health systems are diversified in the three countries, based on organizational hierarchy, human resource quantity and capacity. Further, the compatibility between LPEP and national programs is high, as the existing contact tracing system (including infrastructure and staff) is retained and strengthened. As a result, contact tracing is intensified, but needs to be maintained after LPEP program completion. The ownership of the program lies with the government, and their active involvement increases the chances of integration of SDR into national policies, if the results are promising. The LPEP program has introduced simple but important innovations such as digital information system.

As a limitation, this study summarised the national leprosy programs mainly based on the secondary data. The primary data was collected only at LPEP sites (high endemic), which are small geographical units in the countries. There is a possibility of variation in the activities or intensity of national leprosy programs in other parts of the countries, especially low or medium endemic area.

### Conclusions

We conclude that LPEP approaches can be integrated into different health systems without major structural and personal changes, but provisions are necessary for the additional monitoring needs. In the first year LPEP faced some challenges, but the program overcame these because of the committed attitude of the health care staff and officials. Intensive supervision and training developed the human resource capacity to implement similar programs in the future.

The London Declaration highlighted that strong and committed health systems are essential to achieve the 2020 targets for leprosy and other NTDs [4]. Therefore, all actions at the local or international level should contribute to health system strengthening [25]. Evidence suggests that integration strengthens the general health care systems and also enhances the efficiency and sustainability of the disease specific activities if applied properly [31, 38]. Based on the above principles, LPEP was designed and successfully started its operations in coordination with the respective national programs. The next course of action is to apply the findings of this study, while assessing the impact of LPEP in future.

## Additional files

Additional file 1: Staff Interview Questionnaire for Phase I and II. (DOCX 15 kb)

Additional file 2: The national strategies on leprosy control/elimination adopted by India, Nepal and Indonesia. (DOC 35 kb)

## Abbreviations

DNH: Dadra and Nagar Haveli
IEC: Information Education and Communication
LPEP: Leprosy Post-Exposure Prophylaxis
MDT: Multi-drug therapy
NCDR: New Case Detection Rate
NLCP: National Leprosy Control Program
NLEP: National Leprosy Elimination Program
NTD: Neglected Tropical Diseases
PEP: Post-Exposure Prophylaxis
SDR: Single Dose Rifampicin
UT: Union Territory
WHO: World Health Organization
