# Supplementary material for: Introducing leprosy post-exposure prophylaxis into the health systems of India, Nepal and Indonesia: a case study
Source: BMC Health Serv Res. 2017 Sep 29;17:684. doi: 10.1186/s12913-017-2611-7 (PMC5622547; doi:10.1186/s12913-017-2611-7)
Supplement: Supplementary file 1 — Staff Interview Questionnaire for Phase I and II. (DOCX 15 kb) [file 12913_2017_2611_MOESM1_ESM.docx]

**Additional file 1:** Staff Interview Questionnaire for Phase I and II

**Staff Interview Questionnaire**

| Phase (Tick) | Country | Designation | Date |
| --- | --- | --- | --- |
| - I (National Program - II (LPEP) | - India - Nepal - Indonesia |  |  |

*Instructions:*

*Phase I- Verify the standard operating procedures and data trends published by the national leprosy programs through below questions.*

*Phase II- Probe the difference between planned and actual implementation of LPEP and alignment with national leprosy program*

Q1. What is the current epidemiological trend of leprosy in your area?

Q2. What is the reason for current epidemiological trend of leprosy in your area?

Q3. Please describe the National Leprosy Program (I)/ integrated LPEP practices (II) in your area?

Q4. Please describe the associated challenges with National Leprosy Program (I)/ integrated LPEP practices (II) in your area?

Q5. Please suggest the strategies to overcome said challenges?
