# Supplementary material for: Introducing leprosy post-exposure prophylaxis into the health systems of India, Nepal and Indonesia: a case study
Source: BMC Health Serv Res. 2017 Sep 29;17:684. doi: 10.1186/s12913-017-2611-7 (PMC5622547; doi:10.1186/s12913-017-2611-7)
Supplement: Supplementary file 2 — The national strategies on leprosy control/elimination adopted by India, Nepal and Indonesia. (DOC 35 kb) [file 12913_2017_2611_MOESM2_ESM.doc]

| **1. The national strategies on leprosy control/elimination adopted by India, Nepal and Indonesia** | | |
| --- | --- | --- |
| **NLEP, India** | **NLCP, Nepal** | **NLCP, Indonesia** |
| Early diagnosis & prompt MDT, through routine and special efforts | Early new case detection and their timely and complete management | Early new case detection through active case finding (since 2004) |
| Early detection & complete treatment of new leprosy cases. | Quality leprosy services in an integrated setup by qualified health  workers | Provide quality leprosy services, including rehabilitation services , integrated with primary health care and referral |
| Carrying out house hold contact survey in detection of Multibacillary (MB) & child cases. | Prevention of leprosy associated impairment and disability | Focus on Information, Education & Communication (IEC) |
| Decentralized integrated leprosy services through General Health Care system. | Rehabilitation of people affected by leprosy, including medical and community based  rehabilitation | Focus on Disability Prevention & Medical Rehabilitation (DPMR) services |
| Involvement of Accredited Social Health Activists (ASHAs) in the detection & complete treatment of Leprosy cases for leprosy work | Reduce stigma and discrimination through advocacy, social mobilization and IEC activities and  address gender equality and social inclusion | Incorporation of innovative methods to decrease transmission |
| Strengthening of Disability Prevention & Medical Rehabilitation (DPMR) services. | Strengthen referral centers for complications management |  |
| Information, Education & Communication (IEC) activities in the community to improve  self reporting to Primary Health Centre (PHC) and reduction of stigma. | Meaningful involvement of people affected by leprosy in leprosy services, and address human  right issues |  |
| Intensive monitoring and supervision at Primary Health Centre/Community Health Centre. | Promote and conduct operational researches/studies |  |
|  | Monitoring, supportive supervision including onsite coaching, surveillance and evaluation to ensure/strengthen quality leprosy services |  |
|  | Strengthen partnership, co‐operation and coordination with local government, external  development partners, civil society and community based organizations. |  |
